# More results on Simpson’s type inequality through convexity for twice differentiable continuous mappings

**DOI:** 10.1186/s40064-016-1683-x

**Published:** 2016-01-26

**Authors:** Sabir Hussain, Shahid Qaisar

**Affiliations:** Department of Mathematics, College of Science, Qassim University, P. O. Box 6644, Buraydah, 51452 Saudi Arabia; Department of Mathematics, COMSATS Institute of Information Technology, Sahiwal, Pakistan

**Keywords:** Simpson’s inequality, Strongly s-convex function, Integral identity, Holder’s integral inequality, 26D15, 26E60, 41A55

## Abstract

Our aim in this article is to incorporate the notion of “strongly s-convex function” and prove a new integral identity. Some new inequalities of Simpson type for strongly s-convex function utilizing integral identity and Holder’s inequality are considered.

## Background

The following definition is well known in the literature as convex function:

Let $f:I\subseteq R\rightarrow R$ be a function defined on the interval *I* of real numbers. Then *f* is called convex, if $f\left( {\lambda x+\left( {1-\lambda } \right) y} \right) \le \lambda f\left( x \right) +\left( {1-\lambda } \right) f\left( y \right) ,$ for all $x,y\in I$ and $\lambda \in \left[ {0,1} \right] .$ Geometrically, this means that if P, Q and R are three distinct points on graph of *f* with Q between P and R, then Q is on or below chord PR.

Hudzik and Maligrada (1994) defined *s*-convex function as: A function $f:\left[ {0,\infty } \right) \rightarrow R$ is said to be *s*-convex function in the first sense, if $f\left( {\mu x+\nu y} \right) \le \mu ^{s} f\left( x \right) +\nu ^{s} f\left( y \right)$, for all $x,y\in \left[ {0,\infty } \right)$ and $\mu ,\nu \ge 0,$ with $\mu ^{s} + \nu ^{s} = 1$. We denote this by $K_\alpha ^1$, for some fixed $s \in (0, 1]$. Also $f:\left[ {0,\infty } \right) \rightarrow R$ is said to be *s*-convex function in the second sense, if above condition holds for all $x,y\in \left[ {0,\infty } \right)$ and $\mu ,\nu \ge 0,$ with $\mu + \nu = 1$.

The following is very important and well known in the literature, as Simpson’s inequality:

1 $$\begin{aligned} \left| {\int \limits _a^b {f(x)dx} -\frac{(b-a)}{3}\left[ {\frac{f(a)+f(b)}{2}+2f\left( {\frac{a+b}{2}} \right) } \right] } \right| \le \frac{1}{2880}\left\| {f^{\left( 4 \right) }} \right\| _\infty \left( {b-a} \right) ^5, \end{aligned}$$

where the mapping $f:[a,b]\rightarrow R$ is supposed to be a four times continuously differentiable on the interval $\left( {a,b} \right)$ and having the fourth derivative bounded on $\left( {a,b} \right) ,$ that is

$$\begin{aligned} \left\| {f^{\left( 4 \right) }} \right\| _\infty =\sup { }_{x\in \left( {a,b} \right) }\left| {f^{\left( 4 \right) }\left( x \right) } \right| <\infty . \end{aligned}$$

Dragomir et al. (2000) proved that: Let $f:[a, b] \rightarrow R$ be a differentiable function on $I^0$ (interior of *I*) $a,b\in I$ with $a<b.$ If the mapping $\left| {{f}'} \right|$ is convex on $\left[ {a,b} \right] ,$ then we have the following inequality:

2 $$\begin{aligned} \left| {\frac{f\left( a \right) +f\left( b \right) }{2}-\frac{1}{b-a}\int _a^b {f\left( x \right) dx} } \right| \le \frac{\left( {b-a} \right) \left( {\left| {{f}'\left( a \right) } \right| +\left| {{f}'\left( b \right) } \right| } \right) }{8}. \end{aligned}$$

Sarikaya et al. (2010) showed that: Let $f: [a, b]\rightarrow R$ be a differentiable function on $I^0$(interior of *I*) such that $f^{'} \in L_{1}[a, b]$, where $a,b\in I$ with $a<b.$ If the mapping $\left| {{f}'} \right|$ is s-convex on $\left[ {a,b} \right] ,$ for some fixed $s\in \left( {0,1} \right] ,$ then we have the following inequality

3 $$\begin{aligned}&\left| {\frac{1}{6}\left[ {f(a)+4f\left( {\frac{a+b}{2}} \right) +f(b)} \right] -\frac{1}{b-a}\int \limits _a^b {f(x)dx} } \right| \nonumber \\&\quad \le \frac{\left( {s-4} \right) 6^{s+1}+2\times 5^{s+2}-2\times 3^{s+2}+2}{6^{s+2}\left( {s+1} \right) \left( {s+2} \right) }\left( {b-a} \right) \left( {\left| {{f}'\left( a \right) } \right| +\left| {{f}'\left( b \right) } \right| } \right) . \end{aligned}$$

Alomari et al. (2011) established that: Let $f:I\subset R\rightarrow R$ be a differentiable function on $I^0$ (interior of *I*)$a,b\in I$ with $a<b.$ If the mapping $\left| {{f}'} \right|$ is s-convex on $\left[ {a,b} \right] ,$ for some $s\in \left( {0,1} \right] ,$ then we have the following inequality:

4 $$\begin{aligned}&\left| {f\left( {\frac{a+b}{2}} \right) -\frac{1}{b-a}\int _a^b {f\left( x \right) dx} } \right| \nonumber \\&\quad \le \frac{b-a}{4\left( {s+1} \right) \left( {s+2} \right) }\left\{ {\left| {{f}'\left( a \right) } \right| +\left| {{f}'\left( b \right) } \right| +2\left( {s+1} \right) \left| {f^{'}\left( {\frac{a+b}{2}} \right) } \right| } \right\} \nonumber \\&\quad \le \frac{\left( {2^{2-s}+1} \right) \left( {b-a} \right) }{4\left( {s+1} \right) \left( {s+2} \right) }\left[ {\left| {{f}'\left( a \right) } \right| +\left| {{f}'\left( b \right) } \right| } \right] . \end{aligned}$$

For utilizing different kinds of convexities, additional findings relating to the Simpson’s type inequality, readers are directed to Dragomir et al. (2000), Qaisar et al. (2013), Hussain and Qaisar (2014), Dragomir (1999), Wang et al. (2013), Xi and Qi (2013) and Pearce and Pecari’c (2000).

## Main results

To prove our main result, we need the following definition and lemma.

### **Definition 1**

(*Polyak *1996) Let $f:I\subseteq R\rightarrow R$ is said to be strongly s-convex with modulus $c>0$ and for some fixed $s\in \left( {0,1} \right]$, if

$$\begin{aligned} f\left( {\lambda x+\left( {1-\lambda } \right) y} \right) \le \lambda ^{s} f\left( x \right) +\left( {1-\lambda } \right) ^{s}f\left( y \right) -c\lambda \left( {1-\lambda } \right) \left( {x-y} \right) ^2, \end{aligned}$$

for all $x,y\in I$ and $\lambda \in \left[ {0,1} \right] .$

### **Observation 2**

It is clear that, any strongly s-convex function is a strong convex function but the converse is not true in general.

Now we prove the following lemma:

### **Lemma 3**

*Let *$f: I = \left[ {a,b} \right] \subset R\rightarrow R$*be such that*${f}'$*is absolutely continuous and *${f}''\in L_1 \left( {\left[ {a,b} \right] } \right) .$*Then the following inequality holds:*

5 $$\begin{aligned}&\left| {\frac{1}{6}\left[ {f\left( a \right) +2f\left( {\frac{3a+b}{4}} \right) +2f\left( {\frac{a+3b}{4}} \right) +f\left( b \right) } \right] -\frac{1}{b-a}\int \limits _a^b {f(x)dx} } \right| \le \frac{\left( {b-a} \right) ^2}{96} \nonumber \\&\quad \times \int \limits _0^1 {\psi \left( {1-\psi } \right) \left[ {{f}''\left( {\frac{3+\psi }{4}a+\frac{1-\psi }{4}b} \right) +{f}''\left( {\frac{1+\psi }{4}a+\frac{3-\psi }{4}b} \right) +{f}''\left( {\frac{\psi }{4}a+\frac{4-\psi }{4}b} \right) } \right] } d\psi . \end{aligned}$$

### *Proof*

Using integrating by parts, we have

$$\begin{aligned}&\int \limits _0^1 {\psi \left( {1-\psi } \right) {f}''\left( {\frac{3+\psi }{4}a+\frac{1-\psi }{4}b} \right) d\psi } \\&\quad =-\frac{4}{b-a}\left[ {\psi \left( {1-\psi } \right) \left. {{f}'\left( {\frac{3+\psi }{4}a+\frac{1-\psi }{4}b} \right) } \right| _0^1 -\int \limits _0^1 {\left( {1-2\psi } \right) {f}'\left( {\frac{3+\psi }{4}a+\frac{1-\psi }{4}b} \right) d\psi } } \right] \\&\quad =-\frac{16}{\left( {b-a} \right) ^2}\left[ {\left( {1-2\psi } \right) \left. {f\left( {\frac{3+\psi }{4}a+\frac{1-\psi }{4}b} \right) } \right| _0^1 +2\int \limits _0^1 {f\left( {\frac{3+\psi }{4}a+\frac{1-\psi }{4}b} \right) d\psi } } \right] \\&\quad =\frac{16}{\left( {b-a} \right) ^2}\left[ {f\left( a \right) +f\left( {\frac{3a+b}{4}} \right) -\frac{96}{\left( {b-a} \right) ^3}\int \limits _a^{{\left( {3a+b} \right) } /4} {f\left( x \right) dx} } \right] . \\ \end{aligned}$$

Analogously,

$$\begin{aligned}&\int \limits _0^1 {\psi \left( {1-\psi } \right) {f}''\left( {\frac{1+\psi }{4}a+\frac{3-\psi }{4}b} \right) d\psi } \\&\quad =\frac{16}{\left( {b-a} \right) ^2}\left[ {f\left( {\frac{3a+b}{4}} \right) +f\left( {\frac{a+3b}{4}} \right) } \right] -\frac{96}{\left( {b-a} \right) ^3}\int \limits _{{\left( {3a+b} \right) } / 4}^{{\left( {a+3b} \right) }/4} {f\left( x \right) dx}. \\ \end{aligned}$$

And

$$\begin{aligned}&\int \limits _0^1 {\psi \left( {1-\psi } \right) {f}''\left( {\frac{\psi }{4}a+\frac{4-\psi }{4}b} \right) d\psi } \\&\quad =\frac{16}{\left( {b-a} \right) ^2}\left[ {f\left( {\frac{a+3b}{4}} \right) +f\left( b \right) } \right] -\frac{96}{\left( {b-a} \right) ^3}\int \limits _{{\left( {a+3b} \right) } /4}^b {f\left( x \right) dx}. \end{aligned}$$

This proves as required.

### **Theorem 4**

*Let*$f: I = \left[ {a,b} \right] \subset R\rightarrow R$*be such that *${f}'$*is absolutely continuous and *${f}''\in L_1 \left( {\left[ {a,b} \right] } \right) .$*If the mapping*$\left| {{f}''} \right|$*is strongly s-convex on *$\left[ {a,b} \right] ,$*for*$q\ge 1$*and for some fixed *$s\in \left( {0,1} \right]$*, then we have the following inequality:*

$$\begin{aligned}&\left| {\frac{1}{6}\left[ {f\left( a \right) +2f\left( {\frac{3a+b}{4}} \right) +2f\left( {\frac{a+3b}{4}} \right) +f\left( b \right) } \right] -\frac{1}{b-a}\int \limits _a^b {f(x)dx} } \right| \\&\quad \le \frac{6^{1 / q}\left( {b-a} \right) ^2}{576}\left\{ {\begin{array}{l} \left[ {\begin{array}{l} \frac{\left( {s -5} \right) 4^{s +2}+\left( {s +9} \right) 3^{s +2}}{\left( {s +1} \right) \left( {s +2} \right) \left( {s +3} \right) 4^s }\left| {{f}''\left( a \right) } \right| ^q+\frac{1}{\left( {s +2} \right) \left( {s +3} \right) 4^s }\left| {{f}''\left( b \right) } \right| ^q \\ -\frac{17c\left( {b-a} \right) ^2}{960} \\ \end{array}} \right] ^{1 /q} \\ +\left[ {\begin{array}{l} \frac{\left( {s -1} \right) 2^{s +2}+s +5}{\left( {s +1} \right) \left( {s +2} \right) \left( {s +3} \right) 4^s }\left| {{f}''\left( a \right) } \right| ^q+\frac{\left( {s -3} \right) 3^{s +2}+\left( {s +7} \right) 2^{s +2}}{\left( {s +1} \right) \left( {s +2} \right) \left( {s +3} \right) 4^s }\left| {{f}''\left( b \right) } \right| ^q \\ -\frac{37c\left( {b-a} \right) ^2}{960} \\ \end{array}} \right] ^{1 / q} \\ +\left[ {\frac{1}{\left( {s +2} \right) \left( {s +3} \right) 4^s }\left| {{f}''\left( a \right) } \right| ^q+\frac{\left( {s -5} \right) 4^{s +2}+\left( {s +9} \right) 3^{s +2}}{\left( {s +1} \right) \left( {s +2} \right) \left( {s +3} \right) 4^s }\left| {{f}''\left( b \right) } \right| ^q-\frac{17c\left( {b-a} \right) ^2}{960}} \right] ^{1/q} \end{array}} \right\} .\\ \end{aligned}$$

### *Proof*

Using Lemma 3 and strongly s-convexity of $\left| {{f}''} \right| ^q$, we have

$$\begin{aligned}&\left| {\frac{1}{6}\left[ {f\left( a \right) +2f\left( {\frac{3a+b}{4}} \right) +2f\left( {\frac{a+3b}{4}} \right) +f\left( b \right) } \right] -\frac{1}{b-a}\int \limits _a^b {f(x)dx} } \right| \\&\quad \le \frac{\left( {b-a} \right) ^2}{96}\left[ {\begin{array}{l} \int \limits _0^1 {\psi \left( {1-\psi } \right) \left| {{f}''\left( {\frac{3+\psi }{4}a+\frac{1-\psi }{4}b} \right) } \right| } d\psi \\ +\int \limits _0^1 {\psi \left( {1-\psi } \right) \left| {{f}''\left( {\frac{1+\psi }{4}a+\frac{3-\psi }{4}b} \right) } \right| } d\psi +\int \limits _0^1 {\psi \left( {1-\psi } \right) \left| {{f}''\left( {\frac{\psi }{4}a+\frac{4-\psi }{4}b} \right) } \right| } d\psi \\ \end{array}} \right] \\&\quad \le \frac{\left( {b-a} \right) ^2}{96}\left[ {\int \limits _0^1 {\psi \left( {1-\psi } \right) } d\psi } \right] ^{1-1 / q}\left\{ {\begin{array}{l} \left[ {\int \limits _0^1 {\psi \left( {1-\psi } \right) \left( {\begin{array}{l} \left( {\frac{3+\psi }{4}} \right) ^s \left| {{f}''\left( a \right) } \right| ^q+\left( {\frac{1-\psi }{4}} \right) ^s \left| {{f}''\left( b \right) } \right| ^q \\ -\frac{c\left( {b-a} \right) ^2}{16}\int \limits _0^1 {\psi \left( {1-\psi } \right) ^2\left( {3+\psi } \right) d\psi } \\ \end{array}} \right) } } \right] ^{1 /q} \\ +\left[ {\int \limits _0^1 {\psi \left( {1-\psi } \right) \left( {\begin{array}{l} \left( {\frac{1+\psi }{4}} \right) ^s \left| {{f}''\left( a \right) } \right| ^q+\left( {\frac{3-\psi }{4}} \right) ^s \left| {{f}''\left( b \right) } \right| ^q \\ -\frac{c\left( {b-a} \right) ^2}{16}\int \limits _0^1 {\psi \left( {1-\psi ^2} \right) \left( {3-\psi } \right) d\psi } \\ \end{array}} \right) } } \right] ^{1 / q} \\ +\left[ {\int \limits _0^1 {\psi \left( {1-\psi } \right) \left( {\begin{array}{l} \left( {\frac{\psi }{4}} \right) ^s \left| {{f}''\left( a \right) } \right| ^q+\left( {\frac{4-\psi }{4}} \right) ^s \left| {{f}''\left( b \right) } \right| ^q \\ -\frac{c\left( {b-a} \right) ^2}{16}\int \limits _0^1 {\psi ^2\left( {1-\psi } \right) \left( {4-\psi } \right) d\psi } \\ \end{array}} \right) } } \right] ^{1 /q} \\ \end{array}} \right\} \\&\quad =\frac{6^{1 / q}\left( {b-a} \right) ^2}{576}\left\{ {\begin{array}{l} \left[ {\begin{array}{l} \frac{\left( {s -5} \right) 4^{s +2}+\left( {s +9} \right) 3^{s +2}}{\left( {s +1} \right) \left( {s +2} \right) \left( {s +3} \right) 4^s }\left| {{f}''\left( a \right) } \right| ^q+\frac{1}{\left( {s +2} \right) \left( {s +3} \right) 4^s }\left| {{f}''\left( b \right) } \right| ^q \\ -\frac{17c\left( {b-a} \right) ^2}{960} \\ \end{array}} \right] ^{1 /q} \\ +\left[ {\begin{array}{l} \frac{\left( {s -1} \right) 2^{s +2}+s +5}{\left( {s +1} \right) \left( {s +2} \right) \left( {s +3} \right) 4^s }\left| {{f}''\left( a \right) } \right| ^q+\frac{\left( {s -3} \right) 3^{s +2}+\left( {s +7} \right) 2^{s +2}}{\left( {s +1} \right) \left( {s +2} \right) \left( {s +3} \right) 4^s }\left| {{f}''\left( b \right) } \right| ^q \\ -\frac{37c\left( {b-a} \right) ^2}{960} \\ \end{array}} \right] ^{1 /q} \\ +\left[ {\frac{1}{\left( {s +2} \right) \left( {s +3} \right) 4^s }\left| {{f}''\left( a \right) } \right| ^q+\frac{\left( {s -5} \right) 4^{s +2}+\left( {s +9} \right) 3^{s +2}}{\left( {s +1} \right) \left( {s +2} \right) \left( {s +3} \right) 4^s }\left| {{f}''\left( b \right) } \right| ^q-\frac{17c\left( {b-a} \right) ^2}{960}} \right] ^{1 / q} \\ \end{array}} \right\} . \end{aligned}$$

### **Corollary 5**

*Under the conditions of Theorem *4,

1. If $$q = 1$$, then
  $$\begin{aligned}&\left| {\frac{1}{6}\left[ {f\left( a \right) +2f\left( {\frac{3a+b}{4}} \right) +2f\left( {\frac{a+3b}{4}} \right) +f\left( b \right) } \right] -\frac{1}{b-a}\int \limits _a^b {f(x)dx} } \right| \\&\quad \le \frac{\left( {b-a} \right) ^2}{96}\left[ {\frac{\left( {s -3} \right) 4^{s +2}+\left( {2s +6} \right) \left( {3^{s +2}+1} \right) }{\left( {s +1} \right) \left( {s +2} \right) \left( {s +3} \right) 4^s }\left( {\left| {{f}''\left( a \right) } \right| +\left| {{f}''\left( b \right) } \right| } \right) -\frac{71c\left( {b-a} \right) ^2}{960}} \right] . \end{aligned}$$
2. If $$q = 1$$ and $$s = 1$$, then
  $$\begin{aligned}&\left| {\frac{1}{6}\left[ {f\left( a \right) +2f\left( {\frac{3a+b}{4}} \right) +2f\left( {\frac{a+3b}{4}} \right) +f\left( b \right) } \right] -\frac{1}{b-a}\int \limits _a^b {f(x)dx} } \right| \\&\quad \le \frac{\left( {b-a} \right) ^2}{96}\left[ {\left( {\left| {{f}''\left( a \right) } \right| +\left| {{f}''\left( b \right) } \right| } \right) -\frac{71c\left( {b-a} \right) ^2}{960}} \right] . 
\end{aligned}$$

### **Theorem 6**

*Letf be defined as in Theorem* 4 and the mapping $\left| {{f}''} \right| ^q$*is strongly s-convex on*$\left[ {a,b} \right] ,$*for*$q>1$*and for some fixed*$s\in \left( {0,1} \right]$*, then we have the following inequality:*

$$\begin{aligned}&\left| {\frac{1}{6}\left[ {f\left( a \right) +2f\left( {\frac{3a+b}{4}} \right) +2f\left( {\frac{a+3b}{4}} \right) +f\left( b \right) } \right] -\frac{1}{b-a}\int \limits _a^b {f(x)dx} } \right| \\&\quad \le \frac{\left( {b-a} \right) ^2}{96}\left[ {B\left( {\frac{2q-1}{q-1},\frac{2q-1}{q-1}} \right) } \right] ^{1-1 /q}\left[ {\left( {\frac{1}{4^s \left( {s +1} \right) }} \right) } \right] ^{1/q} \\&\qquad \times \, \left\{ {\begin{array}{l} \left[ {\left( {4^{s +1}-3^{s +1}} \right) \left| {{f}''\left( a \right) } \right| ^q+\left| {{f}''\left( b \right) } \right| ^q-\frac{5c\left( {b-a} \right) ^2\left( {s +1} \right) 4^s }{48}} \right] ^{1/ q} \\ +\left[ {\left( {2^{s +1}-1} \right) \left| {{f}''\left( a \right) } \right| ^q+\left( {3^{s +1}-2^{s +1}} \right) \left| {{f}''\left( b \right) } \right| ^q-\frac{11c\left( {b-a} \right) ^2\left( {s +1} \right) 4^s }{48}} \right] ^{1 /q} \\ +\left[ {\left| {{f}''\left( a \right) } \right| ^q+\left( {4^{s +1}-3^{s +1}} \right) \left| {{f}''\left( b \right) } \right| ^q-\frac{5c\left( {b-a} \right) ^2\left( {s +1} \right) 4^s }{48}} \right] ^{1 /q} \\ \end{array}} \right\} , \end{aligned}$$

where $B\left( {\alpha ,\beta } \right)$ is the classical Beta function which may be defined by

$$\begin{aligned} B\left( {\alpha ,\beta } \right) =\int \limits _0^1 {\psi ^{\alpha -1}\left( {1-\psi } \right) ^{\beta -1}} d\psi ,\quad s ,\beta >0. \end{aligned}$$

### *Proof*

Using Lemma 3, strong s-convexity of $\left| {{f}''} \right| ^q$ and Holder’s inequality, we have

$$\begin{aligned}&\left| {\frac{1}{6}\left[ {f\left( a \right) +2f\left( {\frac{3a+b}{4}} \right) +2f\left( {\frac{a+3b}{4}} \right) +f\left( b \right) } \right] -\frac{1}{b-a}\int \limits _a^b {f(x)dx} } \right| \\&\quad \le \frac{\left( {b-a} \right) ^2}{96}\left[ {\begin{array}{l} \int \limits _0^1 {\psi \left( {1-\psi } \right) \left| {{f}''\left( {\frac{3+\psi }{4}a+\frac{1-\psi }{4}b} \right) } \right| } d\psi \\ +\int \limits _0^1 {\psi \left( {1-\psi } \right) \left| {{f}''\left( {\frac{1+\psi }{4}a+\frac{3-\psi }{4}b} \right) } \right| } d\psi +\int \limits _0^1 {\psi \left( {1-\psi } \right) \left| {{f}''\left( {\frac{\psi }{4}a+\frac{4-\psi }{4}b} \right) } \right| } d\psi \\ \end{array}} \right] \\&\quad \begin{array}{l} \le \frac{\left( {b-a} \right) ^2}{96}\left[ {\int \limits _0^1 {\left[ {\psi \left( {1-\psi } \right) } \right] } ^{q / {\left( {q-1} \right) }}d\psi } \right] ^{1-1 / q}\left\{ {\begin{array}{l} \left[ {\left( {\begin{array}{l} \int \limits _0^1 {\left( {\left( {\frac{3+\psi }{4}} \right) ^s \left| {{f}''\left( a \right) } \right| ^q+\left( {\frac{1-\psi }{4}} \right) ^s \left| {{f}''\left( b \right) } \right| ^q} \right) } \\ -\frac{c\left( {b-a} \right) ^2}{16}\int \limits _0^1 {\left( {1-\psi } \right) \left( {3+\psi } \right) d\psi } \\ \end{array}} \right) } \right] ^{1 /q} \\ +\left[ {\left( {\begin{array}{l} \int \limits _0^1 {\left( {\left( {\frac{1+\psi }{4}} \right) ^s \left| {{f}''\left( a \right) } \right| ^q+\left( {\frac{3-\psi }{4}} \right) ^s \left| {{f}''\left( b \right) } \right| ^q} \right) } \\ -\frac{c\left( {b-a} \right) ^2}{16}\int \limits _0^1 {\left( {1-\psi } \right) \left( {3-\psi } \right) d\psi } \\ \end{array}} \right) } \right] ^{1 /q} \\ +\left[ {\left( {\begin{array}{l} \int \limits _0^1 {\left( {\left( {\frac{\psi }{4}} \right) ^s \left| {{f}''\left( a \right) } \right| ^q+\left( {\frac{4-\psi }{4}} \right) ^s \left| {{f}''\left( b \right) } \right| ^q} \right) } \\ -\frac{c\left( {b-a} \right) ^2}{16}\int \limits _0^1 {\psi \left( {4-\psi } \right) d\psi } \\ \end{array}} \right) } \right] ^{1/q} \\ \end{array}} \right\} \\ \end{array}\\&\qquad \times \begin{array}{l} \left| {\frac{1}{6}\left[ {f\left( a \right) +2f\left( {\frac{3a+b}{4}} \right) +2f\left( {\frac{a+3b}{4}} \right) +f\left( b \right) } \right] -\frac{1}{b-a}\int \limits _a^b {f(x)dx} } \right| \\ \le \frac{\left( {b-a} \right) ^2}{96}\left[ {B\left( {\frac{2q-1}{q-1},\frac{2q-1}{q-1}} \right) } \right] ^{1-1/q}\left[ {\left( {\frac{1}{4^s \left( {s +1} \right) }} \right) } \right] ^{1/q} \\ \end{array}\\&\qquad \times \, \left\{ {\begin{array}{l} \left[ {\left( {4^{s +1}-3^{s +1}} \right) \left| {{f}''\left( a \right) } \right| ^q+\left| {{f}''\left( b \right) } \right| ^q-\frac{5c\left( {b-a} \right) ^2\left( {s +1} \right) 4^s }{48}} \right] ^{1/q} \\ +\left[ {\left( {2^{s +1}-1} \right) \left| {{f}''\left( a \right) } \right| ^q+\left( {3^{s +1}-2^{s +1}} \right) \left| {{f}''\left( b \right) } \right| ^q-\frac{11c\left( {b-a} \right) ^2\left( {s +1} \right) 4^s }{48}} \right] ^{1 /q} \\ +\left[ {\left| {{f}''\left( a \right) } \right| ^q+\left( {4^{s +1}-3^{s +1}} \right) \left| {{f}''\left( b \right) } \right| ^q-\frac{5c\left( {b-a} \right) ^2\left( {s +1} \right) 4^s }{48}} \right] ^{1 /q} \end{array}} \right\} . \end{aligned}$$

This completes the proof.

### **Theorem 7**

*Let f be defined as in Theorem *4*and the mapping *$\left| {{f}''} \right| ^q$*is strongly s-convex on*$\left[ {a,b} \right] ,$*for*$q>1$*and for some fixed*$s\in \left( {0,1} \right]$*, then we have the following inequality:*

$$\begin{aligned}&\left| {\frac{1}{6}\left[ {f\left( a \right) +2f\left( {\frac{3a+b}{4}} \right) +2f\left( {\frac{a+3b}{4}} \right) +f\left( b \right) } \right] -\frac{1}{b-a}\int \limits _a^b {f(x)dx} } \right| \\&\quad \le \frac{\left( {b-a} \right) ^2}{96}\left[ {\frac{\left( {q-1} \right) ^2}{\left( {2q-1} \right) \left( {3q-2} \right) }} \right] ^{1-1 / q}\left[ {\left( {\frac{1}{4^s \left( {s +1} \right) \left( {s +2} \right) }} \right) } \right] ^{1/ q} \\&\qquad \times \,\left\{ {\begin{array}{l} \left[ {\left( {\left( {s -2} \right) 4^{s +1}+3^{s +2}} \right) \left| {{f}''\left( a \right) } \right| ^q+\left| {{f}''\left( b \right) } \right| ^q-\frac{7c\left( {b-a} \right) ^2\left( {s +1} \right) \left( {s +2} \right) 4^s }{192}} \right] ^{1/ q} \\ +\left[ {\left( {2^{s +1}s +1} \right) \left| {{f}''\left( a \right) } \right| ^q+\left( {3^{s +2}-2^{s +1}\left( {s +4} \right) } \right) \left| {{f}''\left( b \right) } \right| ^q-\frac{23c\left( {b-a} \right) ^2\left( {s +1} \right) \left( {s +2} \right) 4^s }{192}} \right] ^{1 / q} \\ +\left[ {\left( {s +1} \right) \left| {{f}''\left( a \right) } \right| ^q+\left( {4^{s +2}-3^{s +1}\left( {s +5} \right) } \right) \left| {{f}''\left( b \right) } \right| ^q-\frac{13c\left( {b-a} \right) ^2\left( {s +1} \right) \left( {s +2} \right) 4^s }{192}} \right] ^{1/ q} \\ \end{array}} \right\} . \\ \end{aligned}$$

### *Proof*

Using Lemma 3, Holder’s inequality and strongly s- convexity of $\left| {{f}''} \right| ^q$, we have

$$\begin{aligned}&\left| {\frac{1}{6}\left[ {f\left( a \right) +2f\left( {\frac{3a+b}{4}} \right) +2f\left( {\frac{a+3b}{4}} \right) +f\left( b \right) } \right] -\frac{1}{b-a}\int \limits _a^b {f(x)dx} } \right| \\&\quad \le \frac{\left( {b-a} \right) ^2}{96}\left[ {\begin{array}{l} \int \limits _0^1 {\psi \left( {1-\psi } \right) \left| {{f}''\left( {\frac{3+\psi }{4}a+\frac{1-\psi }{4}b} \right) } \right| } d\psi \\ +\int \limits _0^1 {\psi \left( {1-\psi } \right) \left| {{f}''\left( {\frac{1+\psi }{4}a+\frac{3-\psi }{4}b} \right) } \right| } d\psi +\int \limits _0^1 {\psi \left( {1-\psi } \right) \left| {{f}''\left( {\frac{\psi }{4}a+\frac{4-\psi }{4}b} \right) } \right| } d\psi \\ \end{array}} \right] \\&\quad \le \frac{\left( {b-a} \right) ^2}{96}\left[ {\int \limits _0^1 {\left[ {\psi \left( {1-\psi } \right) } \right] } ^{q / {\left( {q-1} \right) }}d\psi } \right] ^{1-1/ q}\left\{ {\begin{array}{l} \left[ {\left( {\begin{array}{l} \int \limits _0^1 {\psi \left( {\left( {\frac{3+\psi }{4}} \right) ^s \left| {{f}''\left( a \right) } \right| ^q+\left( {\frac{1-\psi }{4}} \right) ^s \left| {{f}''\left( b \right) } \right| ^q} \right) } \\ -\frac{c\left( {b-a} \right) ^2}{16}\int \limits _0^1 {\psi \left( {1-\psi } \right) \left( {3+\psi } \right) d\psi } \\ \end{array}} \right) } \right] ^{1 / q} \\ +\left[ {\left( {\begin{array}{l} \int \limits _0^1 {\psi \left( {\left( {\frac{1+\psi }{4}} \right) ^s \left| {{f}''\left( a \right) } \right| ^q+\left( {\frac{3-\psi }{4}} \right) ^s \left| {{f}''\left( b \right) } \right| ^q} \right) } \\ -\frac{c\left( {b-a} \right) ^2}{16}\int \limits _0^1 {\psi \left( {1+\psi } \right) \left( {3-\psi } \right) d\psi } \\ \end{array}} \right) } \right] ^{1 / q} \\ +\left[ {\left( {\begin{array}{l} \int \limits _0^1 {\psi \left( {\left( {\frac{\psi }{4}} \right) ^s \left| {{f}''\left( a \right) } \right| ^q+\left( {\frac{4-\psi }{4}} \right) ^s \left| {{f}''\left( b \right) } \right| ^q} \right) } \\ -\frac{c\left( {b-a} \right) ^2}{16}\int \limits _0^1 {\psi ^2\left( {4-\psi } \right) d\psi } \\ \end{array}} \right) } \right] ^{1 / q} \\ \end{array}} \right\} . \\ \end{aligned}$$

or

$$\begin{aligned}&\left| {\frac{1}{6}\left[ {f\left( a \right) +2f\left( {\frac{3a+b}{4}} \right) +2f\left( {\frac{a+3b}{4}} \right) +f\left( b \right) } \right] -\frac{1}{b-a}\int \limits _a^b {f(x)dx} } \right| \\&\le \frac{\left( {b-a} \right) ^2}{96}\left[ {\frac{\left( {q-1} \right) ^2}{\left( {2q-1} \right) \left( {3q-2} \right) }} \right] ^{1-1 / q}\left[ {\left( {\frac{1}{4^s \left( {s +1} \right) \left( {s +2} \right) }} \right) } \right] ^{1/q} \\&\quad \times \, \left\{ {\begin{array}{l} \left[ {\left( {\left( {s -2} \right) 4^{s +1}+3^{s +2}} \right) \left| {{f}''\left( a \right) } \right| ^q+\left| {{f}''\left( b \right) } \right| ^q-\frac{7c\left( {b-a} \right) ^2\left( {s +1} \right) \left( {s +2} \right) 4^s }{192}} \right] ^{1 / q} \\ +\left[ {\left( {2^{s +1}s +1} \right) \left| {{f}''\left( a \right) } \right| ^q+\left( {3^{s +2}-2^{s +1}\left( {s +4} \right) } \right) \left| {{f}''\left( b \right) } \right| ^q-\frac{23c\left( {b-a} \right) ^2\left( {s +1} \right) \left( {s +2} \right) 4^s }{192}} \right] ^{1/ q} \\ +\left[ {\left( {s +1} \right) \left| {{f}''\left( a \right) } \right| ^q+\left( {4^{s +2}-3^{s +1}\left( {s +5} \right) } \right) \left| {{f}''\left( b \right) } \right| ^q-\frac{13c\left( {b-a} \right) ^2\left( {s +1} \right) \left( {s +2} \right) 4^s }{192}} \right] ^{1 / q} \\ \end{array}} \right\} \end{aligned}$$

This completes the proof.

### **Theorem 8**

*Letfbe defined as in Theorem* 4*and the mapping *$\left| {{f}''} \right| ^q$*is strongly s-convex on *$\left[ {a,b} \right] ,$*for*$q>1$*and for some fixed*$s\in \left( {0,1} \right]$*, then we have the following inequality:*

$$\begin{aligned}&\left| {\frac{1}{6}\left[ {f\left( a \right) +2f\left( {\frac{3a+b}{4}} \right) +2f\left( {\frac{a+3b}{4}} \right) +f\left( b \right) } \right] -\frac{1}{b-a}\int \limits _a^b {f(x)dx} } \right| \\&\quad \le \frac{\left( {b-a} \right) ^2}{96}\left[ {\frac{\left( {q-1} \right) ^2}{\left( {2q-1} \right) \left( {3q-2} \right) }} \right] ^{1-1/ q}\left[ {\left( {\frac{1}{4^s \left( {s +1} \right) \left( {s +2} \right) }} \right) } \right] ^{1 /q} \\&\qquad \times \, \left\{ {\begin{array}{l} \left[ {\left( {4^{s +2}-\left( {s +5} \right) 3^{s +1}} \right) \left| {{f}''\left( a \right) } \right| ^q+\left( {s +1} \right) \left| {{f}''\left( b \right) } \right| ^q-\frac{13c\left( {b-a} \right) ^2\left( {s +1} \right) \left( {s +2} \right) 4^s }{192}} \right] ^{1/q} \\ +\left[ {\left( {2^{s +2}-s -3} \right) \left| {{f}''\left( a \right) } \right| ^q+\left( {3^{s +1}\left( {s -1} \right) -2^{s +2}} \right) \left| {{f}''\left( b \right) } \right| ^q-\frac{21c\left( {b-a} \right) ^2\left( {s +1} \right) \left( {s +2} \right) 4^s }{192}} \right] ^{1/q} \\ +\left[ {\left| {{f}''\left( a \right) } \right| ^q+\left( {\left( {s -2} \right) 4^{s +1}+3^{s +2}} \right) \left| {{f}''\left( b \right) } \right| ^q-\frac{7c\left( {b-a} \right) ^2\left( {s +1} \right) \left( {s +2} \right) 4^s }{192}} \right] ^{1/ q} \\ \end{array}} \right\} . \end{aligned}$$

### *Proof*

Using Lemma 3, Holder inequality and strongly s- convexity of $\left| {{f}''} \right|$, we have

$$\begin{aligned}&\left| {\frac{1}{6}\left[ {f\left( a \right) +2f\left( {\frac{3a+b}{4}} \right) +2f\left( {\frac{a+3b}{4}} \right) +f\left( b \right) } \right] -\frac{1}{b-a}\int \limits _a^b {f(x)dx} } \right| \\&\quad \le \frac{\left( {b-a} \right) ^2}{96}\left[ {\begin{array}{l} \int \limits _0^1 {\psi \left( {1-\psi } \right) \left| {{f}''\left( {\frac{3+\psi }{4}a+\frac{1-\psi }{4}b} \right) } \right| } d\psi \\ +\int \limits _0^1 {\psi \left( {1-\psi } \right) \left| {{f}''\left( {\frac{1+\psi }{4}a+\frac{3-\psi }{4}b} \right) } \right| } d\psi +\int \limits _0^1 {\psi \left( {1-\psi } \right) \left| {{f}''\left( {\frac{\psi }{4}a+\frac{4-\psi }{4}b} \right) } \right| } d\psi \\ \end{array}} \right] \\&\quad \le \frac{\left( {b-a} \right) ^2}{96}\left[ {\int \limits _0^1 {\left( {1-\psi } \right) } \psi ^{q /{\left( {q-1} \right) }}d\psi } \right] ^{1-1 / q}\left\{ {\begin{array}{l} \left[ {\left( {\begin{array}{l} \int \limits _0^1 {\left( {1-\psi } \right) \left( {\left( {\frac{3+\psi }{4}} \right) ^s \left| {{f}''\left( a \right) } \right| ^q+\left( {\frac{1-\psi }{4}} \right) ^s \left| {{f}''\left( b \right) } \right| ^q} \right) } \\ -\frac{c\left( {b-a} \right) ^2}{16}\int \limits _0^1 {\left( {1-\psi } \right) ^2\left( {3+\psi } \right) d\psi } \\ \end{array}} \right) } \right] ^{1 / q} \\ +\left[ {\left( {\begin{array}{l} \int \limits _0^1 {\left( {1-\psi } \right) \left( {\left( {\frac{1+\psi }{4}} \right) ^s \left| {{f}''\left( a \right) } \right| ^q+\left( {\frac{3-\psi }{4}} \right) ^s \left| {{f}''\left( b \right) } \right| ^q} \right) } \\ -\frac{c\left( {b-a} \right) ^2}{16}\int \limits _0^1 {\left( {1-\psi ^2} \right) \left( {3-\psi } \right) d\psi } \\ \end{array}} \right) } \right] ^{1 / q} \\ +\left[ {\left( {\begin{array}{l} \int \limits _0^1 {\left( {1-\psi } \right) \left( {\left( {\frac{\psi }{4}} \right) ^s \left| {{f}''\left( a \right) } \right| ^q+\left( {\frac{4-\psi }{4}} \right) ^s \left| {{f}''\left( b \right) } \right| ^q} \right) } \\ -\frac{c\left( {b-a} \right) ^2}{16}\int \limits _0^1 {\psi \left( {1-\psi } \right) \left( {4-\psi } \right) d\psi } \\ \end{array}} \right) } \right] ^{1 / q} \\ \end{array}} \right\} . \\ \end{aligned}$$

or

$$\begin{aligned}&\left| {\frac{1}{6}\left[ {f\left( a \right) +2f\left( {\frac{3a+b}{4}} \right) +2f\left( {\frac{a+3b}{4}} \right) +f\left( b \right) } \right] -\frac{1}{b-a}\int \limits _a^b {f(x)dx} } \right| \\&\quad \le \frac{\left( {b-a} \right) ^2}{96}\left[ {\frac{\left( {q-1} \right) ^2}{\left( {2q-1} \right) \left( {3q-2} \right) }} \right] ^{1-1/ q}\left[ {\left( {\frac{1}{4^s \left( {s +1} \right) \left( {s +2} \right) }} \right) } \right] ^{1/ q} \\&\qquad \times \, \left\{ {\begin{array}{l} \left[ {\left( {4^{s +2}-\left( {s +5} \right) 3^{s +1}} \right) \left| {{f}''\left( a \right) } \right| ^q+\left( {s +1} \right) \left| {{f}''\left( b \right) } \right| ^q-\frac{13c\left( {b-a} \right) ^2\left( {s +1} \right) \left( {s +2} \right) 4^s }{192}} \right] ^{1 / q} \\ +\left[ {\left( {2^{s +2}-s -3} \right) \left| {{f}''\left( a \right) } \right| ^q+\left( {3^{s +1}\left( {s -1} \right) -2^{s +2}} \right) \left| {{f}''\left( b \right) } \right| ^q-\frac{21c\left( {b-a} \right) ^2\left( {s +1} \right) \left( {s +2} \right) 4^s }{192}} \right] ^{1/ q} \\ +\left[ {\left| {{f}''\left( a \right) } \right| ^q+\left( {\left( {s -2} \right) 4^{s +1}+3^{s +2}} \right) \left| {{f}''\left( b \right) } \right| ^q-\frac{7c\left( {b-a} \right) ^2\left( {s +1} \right) \left( {s +2} \right) 4^s }{192}} \right] ^{1 /q} \\ \end{array}} \right\} . \end{aligned}$$

This completes the proof. $\square$

## Conclusion

We incorporated notion of “strongly s-convex function” and proved a new integral identity. Some new inequalities of Simpson type for strongly s-convex function utilizing integral identity and Holder’s inequality are also considered. These results give better estimates as presented earlier in the literature.
